# The validity of a food frequency questionnaire as a measure of PUFA status in pregnancy

**DOI:** 10.1186/s12884-015-0494-3

**Published:** 2015-03-18

**Authors:** Gordon Parker, Georgia McClure, Bronwyn D Hegarty, Isabelle Granville Smith

**Affiliations:** School of Psychiatry, University of New South Wales, Sydney, NSW Australia; Black Dog Institute, Hospital Rd, Randwick, 2031 NSW Australia

**Keywords:** Food frequency questionnaire, Polyunsaturated fatty acid, Biomarkers, Pregnancy

## Abstract

**Background:**

Nutritional studies have found conflicting evidence regarding the ability of Food Frequency Questionnaires (FFQs) to demonstrate convergent validity with tissue content of omega-3 and omega-6 polyunsaturated fatty acids (PUFAs). We therefore sought to assess the convergent validity of a FFQ strategy when compared with a blood biomarker of PUFA levels in a sample of pregnant women.

**Method:**

A previously validated PUFA FFQ was completed by 895 pregnant women and compared to erythrocyte membrane of six PUFA variables.

**Results:**

Four of the six correlations were found to be formally significant, however two of these demonstrated minimal associational strength. Moderate-high correlations between the FFQ-derived PUFA intake estimates and blood biomarker PUFA levels were shown only for eicosapentaenoic acid (EPA; 0.55) and docosahexaenoic acid (DHA; 0.61).

**Conclusions:**

Overall, the correlations were lower than those found in general population studies. Findings suggest biological estimates, such as blood samples, may be most appropriate to measure PUFA levels above indirect strategies such as an FFQ in this population. The results, if an indirect strategy is unavoidable, indicate specific PUFAs where an FFQ strategy may be most informative.

## Background

Several omega-3 polyunsaturated fatty acids (PUFAs) are considered important for normal neuronal development and function [1]. Research suggests a possible antidepressant role of omega-3 supplementation [2], and as providing benefit for some inflammatory disorders [3]. While supplementation is advocated for many conditions, empirical support is variable. Although suggested as protective against cognitive decline, a systematic review [4] established no benefit of omega-3 supplements in cognitively-healthy older adults. Whilst some studies suggest omega-3 supplements may reduce cardiac risk [5-7] a recent systematic review concluded that, while high circulating omega-3 is associated with reduced cardiovascular events, supplementation appears to have no cardiovascular benefit [8]. Preliminary evidence of benefit has also been described for neuropsychiatric disorders such as autism and attention deficit hyperactivity disorder [9].

As omega-3 PUFAs are increasingly positioned as having a broad therapeutic role, efficacy studies are likely to increase. In such evaluations, a clear advantage is that PUFAs can be measured directly in red blood cells (RBCs), plasma and adipose tissue, allowing quantitative rigor. However, it is not uncommon for evaluative studies to measure PUFA levels indirectly, and most commonly by food frequency questionnaires (FFQs) that retrospectively seek details on frequency of consumption as well as portion size of foods and nutrients over a specific period [10]. FFQs are suggested useful for nutrients such as PUFAs, as daily intake tend to vary [11]. FFQ proponents argue their utility on grounds of low participant burden, cost-effectiveness and administrative ease [12]. A key issue, however, is their validity. Indirect measures of nutrient intake risk multiple sources of error, relying on accurate memory, as well as reporting and dietary consistency. Accuracy of FFQs may be further compromised by a tendency to under-report consumption, and/or change consumption habits if the participants are aware their dietary intake will be the focus of an upcoming research study (or medical investigation) [13]. Absorption of some fats may vary between individuals from 68% to 94%, and diets high in carbohydrate and saturated fat may alter PUFA absorption [13].

FFQ PUFA studies evaluate both ‘parent’ (omega-3 alpha-linolenic acid (ALA) and omega-6 linoleic acid) and long-chain PUFAs including eicosapentaenoic acid (EPA, omega-3), docosapentaenoic acid (DPA, omega-3), docosahexaenoic acid (DHA, omega-3) and arachidonic acid (AA, omega-6). Of these, EPA and DHA are considered the key omega-3 molecules affecting membrane biophysics, inflammatory processes and gene regulation pathways [1-7].

One systematic review reported FFQ estimates as ‘good’ or ‘acceptable’ (correlations with blood or subcutaneous lipids ranged from 0.40 to 0.60), and on par with other indirect dietary measurements such as dietary history questionnaires and 24-hour recalls [14].

Recent Australian studies have quantified links between FFQ-derived intake data and plasma or RBC PUFA levels. Initial correlations between FFQ intake and omega-3 PUFA levels (with the exception of DHA) in both RBC and plasma biomarkers ranged between 0.39 and 0.54 [15]. This particular FFQ was electronically updated as a computer based, online tool to allow for automatic calculations of estimated daily PUFA intake [16]. The updated FFQ intake estimates showed improved correlations with erythrocyte and plasma content for EPA (with correlations of 0.92 and 0.87), DHA (0.69 and 0.64) and total omega-3 PUFAs (0.78 and 0.48). While encouraging, these validation studies comprised relatively small samples of 53 and 48 participants [15,16].

Given the interest in PUFAs for the management of pre- and post-natal depression [17] - reflecting concerns about use of formal antidepressant medications - it is important to determine whether an FFQ strategy is valid in pregnant women. Previous studies of pregnant women have quantified FFQ PUFA correlations of 0.23 with gluteal adipose tissue stores [18], 0.35 for DHA and 0.36 for EPA with erythrocyte PUFA levels [19], and 0.32 for DHA but not significant for EPA with breast milk PUFA measures [20]. Therefore, this study was undertaken to evaluate whether a FFQ strategy is a valid PUFA measure when compared with RBC PUFA levels in a large sample of pregnant Australian women.

## Method

### Participants

We recruited women between 34 and 37 weeks of pregnancy attending an obstetric service within the Sydney and Central Coast regions of NSW. To meet inclusion criteria for the study participants had to be over 18 years of age, proficient in English and provide written and signed consent. The study was formally approved by the University of New South Wales Human Research Ethics Committee (reference number: 09/CRGH/168) in accordance with the declaration of Helsinki.

### FFQ Data

The 38-item PUFA FFQ developed by Swirk et al. [16] assesses ‘usual’ dietary intake over the previous three months. This FFQ includes a broad range of foods including meat, fish/seafood, eggs, cereals and desserts. Food products fortified with omega-3 and supplements are also included. This particular FFQ was designed for use in studies of healthy adult Australians [12,16] and found to be a valid tool to measure PUFA levels in this population. Participants are asked to report consumption frequency, ranging from ‘never’ up to ‘twice daily or more’. Participants completed a pen and paper version of the questionnaire, which was then entered into the electronic database by a research assistant.

### Blood samples

Details of our blood sampling and analyses are provided elsewhere [21]. Blood samples were collected according to the method of Magunsardottir and Skuladottir [22] in order to limit the effects of oxidation upon membrane lipids. Blood samples were obtained from non-fasting participants, and a dedicated sample was collected specifically for the purpose of the primary study [21]. Where possible the blood was collected alongside routine prenatal appointments with the midwife and, for that reason, time of day for collection was not standardized across the sample.

Briefly, blood was collected in EDTA vaccutainers. Erythrocytes were pelleted, washed in saline and reconstituted in butylated hydroxytoluene (50 μg/ml) for storage at −80°C until analysis. The fatty acid profile of erythrocyte membrane phospholipids was measured at the University of Adelaide. Total lipids were extracted from erythrocytes with chloroform/isopropanol and the phospholipids separated from total lipid extracts by thin layer chromatography. Phospholipids were then transesterified to fatty acid methyl esters and separated and quantified by gas chromatography.

FFQ-quantified estimates were then correlated with erythrocyte-derived levels of total omega-3, total omega-6, ALA, EPA, DPA and DHA. As oxidisation of blood samples may confound analyses, we excluded samples containing a percentage of saturated fatty acids more than two standard deviations above the mean.

### Statistical analysis

Data were analysed via Spearman correlations between erythrocyte PUFA levels and the self-reported PUFA levels in the FFQ. Correlations were defined as significant if *p* < 0.01.

## Results

### Sample features

After excluding oxidised samples, we analysed data for 895 women. Their mean age was 31.0 (5.7) years, 85.4% principally spoke English, 48.0% attained a university qualification, 12.0% a post-graduate degree and 45.3% had a family income above $83,000, indicating higher than average socio-economic backgrounds, 14% reported a significant medical condition although specific conditions were not recorded. Table 1 details the demographic information for this sample.

**Table 1:** **Participant demographics**

**Variables**		**Percentage %**
Age (mean,sd)		31.0, 5.7
English as second language	No	85.4%
	Yes	14.6%
Marital status	Never married	7.3%
	Married	65.7%
	Defacto	24.9%
	Separated	0.9%
	Widowed	0.1%
	Divorced	1.0%
Level of education achieved	Primary School	0.4%
	Some High School	13.4%
	Completed High School	26.2%
	Diploma/Advanced Diploma	15.2%
	Bachelor Degree	26.0%
	Graduate Diploma/Graduate Certificate	6.8%
	Postgraduate Degree	12.0%
Level of family income	Less than $39,000	17.8%
	$39,000-$83,000	36.9%
	More than $83, 0000	45.3%
Reported a significant medical condition	No	86.0%
	Yes	14.0%

### FFQ and erythrocyte phospholipid analyses

Table 2 reports median PUFA FFQ-estimated intakes and median quantified erythrocyte PUFA levels and their correlation. All fatty acid values are expressed as the percentage of total fatty acids in erythrocyte phospholipids. While four of the six correlations were formally significant, the large sample size risked Type I errors. Examining the correlation coefficients, two PUFAs (DHA and EPA) showed moderate to high correlations (0.61 and 0.55 respectively), while the remainder (ranging from +0.14 to −0.02) showed virtually no associational strength.

**Table 2:** **Mean erythrocyte and mean FFQ estimate of differing PUFAs and levels of agreement between each estmate**

**PUFA**	**Erythrocyte** ^**†**^ **(Median/Range)**	**FFQ (Median/Range) intake (g/d)**	**Spearman correlation coefficent**
ALA	0.17 (0.3)	0.02 (0.6)	−0.02
EPA	0.46 (3.8)	0.06 (3.8)	0.55*
DPA	2.27 (3.7)	0.01 (0.2)	0.14*
DHA	6.05 (8.3)	0.15 (1.9)	0.61*
Total Omega-3	9.02 (15.4)	1.66 (12.9)	0.14*
Total Omega-6	27.13 (14.8)	10.52 (82.85)	0.02

*Significant at 0.01.

^†^All fatty acid values are expressed as the percentage of total fatty acids in erythrocyte phospholipids.

Acronyms: PUFA- polyunsaturated fatty acids; FFQ- food frequency questionnaire; ALA- alpha-linolenic acide; EPA- eicosapentataenoic acid; DPA- docosapentaenic acid; DHA- docosahexaenoic acid.

## Discussion

Of the PUFA variables measured, we quantified strong associations between FFQ intake estimates and erythrocyte membrane content of the two key long-chain omega-3 PUFAs, DHA and EPA. Of the other PUFA variables measured ALA, DPA, total omega-3 and total omega-6 PUFA did not show clinically significant associational strength (correlation coefficents below .2).

Our correlations for DHA and EPA erythrocyte levels and FFQ estimations are lower than those previously reported in more general population samples [14,16] but higher than those reported in a study of pregnant women [19] where moderate correlations between FFQ scores and RBC PUFA levels of 0.36 (EPA) and 0.35 (DHA) were found.

Poor correlations between intake estimates and RBC content of ALA and DPA has been previously reported [16], however it is unclear why correlations for total omega-3 and omega-6 PUFA were much lower in the current study compared to previous reports [16,20]. It is possible that estimation of RBC omega-3 status through the indirect measure of dietary intake such as a FFQ may be less valid among pregnant women. Pregnancy may be associated with fluctuating reserves of omega-3 PUFA throughout the different stages of foetal development [23].

Several studies have highlighted that differences in omega-3 PUFA levels in blood samples of pregnant women cannot be explained by modifications in dietary intake alone [24,25]. It is also possible that certain biomarkers may be less reliable in determining PUFA levels in pregnant women due to an increase in maternal plasma volume [26]. As a consequence, several studies have compared FFQ estimates to alternative biomarkers with varied results; moderate correlations were observed between FFQ estimates and adipose tissue as a biomarker [18], and breast milk DHA content showed a moderate correlation (coefficient of 0.32) with FFQ estimates [20]. Future research could extend upon the current study by determining which biomarker is the most reliable to assess PUFA levels during pregnancy.

## Conclusions

In conclusion, correlations between FFQ intake estimates and RBC content of omega-3 and omega-6 PUFA were quantified as lower in this study of a large population of pregnant women compared to previous smaller general population studies. However, strong correlations between intake estimates and RBC biomarker were demonstrated for the key long-chain omega-3 PUFA, EPA and DHA. Such findings argue for researchers to prioritise biological estimates above an indirect strategy such as use of FFQs but, if a default FFQ option is unavoidable, indicate the domains where estimates may have some informative capacity.

## Abbreviations

PUFAs: Polyunsaturated fatty acids
FFQ: Food frequency questionnaire
ALA: Alpha-linolenic acid
EPA: Eicosapentaenoic acid
DPA: Docosapentaenic acid
DHA: Docosahexaenoic acid
AA: Arachidonic Acid
RBC: Red blood cells
